# Investigation of the Degradation Mechanism of SiC MOSFET Subjected to Multiple Stresses

**DOI:** 10.3390/mi14071469

**Published:** 2023-07-21

**Authors:** Huifen Dong, Yunxia Wu, Chan Li, Hai Xu

**Affiliations:** 1 College of Electronic Information and Automation, Civil Aviation University of China, Tianjin 300300, China; 2 Tianjin Aviation Equipment Safety and Airworthiness Technology Innovation Center, Tianjin 300300, China; 3 Shenyang Aircraft Airworthiness Certification Center of CAAC, Shenyang 110043, China

**Keywords:** finite element analysis (FEA), multi-stress, SiC MOSFET

## Abstract

The performance requirements for power devices in airborne equipment are increasingly demanding, while environmental and working stresses are becoming more diverse. The degradation mechanisms of devices subjected to multiple stresses become more complex. Most proposed degradation mechanisms and models in current research only consider a single stress, making it difficult to describe the correlation between multiple stresses and the correlation of failures. Then, a multi-physical field coupling model based on COMSOL is proposed. The influence relationship between temperature, moisture, electrical load, and vibration during device operation is considered, and a three-dimensional finite element model is built to investigate the multi-stress degradation mechanism under multi-physical field coupling. The simulation results show that, compared with single-stress models, the proposed multi-stress coupled model can more accurately simulate the degradation process of SiC MOSFET. This provides references for improving the reliability design of power device packaging.

## 1. Introduction

The electric aircraft power system comprises many power converters, and as a vital component, the reliability of power devices directly determines the safety of the power system. Power devices operate in a highly complex environment that involves temperature, moisture, vibration, etc. Under the combined effects of these multiple stresses, the degradation mechanism of power devices is no longer single.

Numerous scholars have studied the degradation mechanism of electronic components and obtained valuable results. Haojie Wang et al. studied the effect of random vibration on component structure and suggested that increasing substrate thickness can extend device lifespan [1]. Mayssam Jannoun et al. focused on reliability solder joint reliability under random vibration [2]. JiaJia Guan et al. further considered the response of power devices to random vibration under thermal loads and found that thermal stress can affect the structural stiffness and thus stress under vibration [3]. Pushpa Rajaguru assessed solder layer reliability under thermal and vibrational loads and concluded that vibration-induced damage surpasses damage from thermomechanical fatigue at critical resonant frequencies [4]. Xinlan Hu et al. used finite element simulation to study the combined effects of electrical–thermal loads and random vibrations on solder joint reliability [5]. In addition to random vibration, thermal stress has been a widely studied phenomenon [6,7,8,9,10] resulting in bond wire dislodgement and damage to solder layers. Recent attention has also been given to moisture reliability. Zhao Yajun et al. noted that delamination in devices can occur under high-pressure conditions caused by high-temperature wet air diffusion owing to the mismatch in coefficient of thermal expansion (CTE) between the plastic packaging material, chip, and frame [11]. M. H. Wang analyzed moisture diffusion and warpage stress under thermal–moisture coupling loads and concluded that moisture concentration affects passivation layer expansion [12]. Felix Hoffmann et al. investigated the effect of thermo-mechanical stress on device moisture resistance [13], while Yanhao Wang considered the coupling of thermal–moisture multi-physical fields, further analyzed the effect of ambient temperature on relative moisture [14], and then analyzed the influence of moisture on the power cycle life of the device through experiments [15].

Airborne power devices are often characterized by rapid temperature changes and high stress. Their working is affected by both aspects of the natural environment, such as temperature and moisture, as well as the operating environment, such as temperature, electrical load, and vibration. Most degradation mechanisms and models proposed in current research only apply to single stress, which fails to describe the correlation between multiple stresses and failures under multiple stresses. Based on the above, this paper establishes a theoretical and finite element analysis model of power devices under multiple stresses, considering the coupling relationship between temperature, moisture, random vibration, and electrical load. The proposed model enables us to comprehensively analyze the influence of various factors on power device degradation and, more accurately, to describe the degradation process. It can provide references for the packaging design of the device.

## 2. Mathematical Modeling of Inter-Field Coupling for SiC MOSFET

This research focuses on a SiC MOSFET transistor and is based on several assumptions, including:

1. The device is uniformly and securely soldered at each package layer, remaining flat and free from warping in its initial state.
2. Under the influence of temperature and moisture, the device expands uniformly in an isotropic manner.
3. Some material properties of each package layer are invariable constants.

Using the principles of energy conservation, current conservation, generalized Hooke’s law, and Fick’s mass transfer law, a mathematical model is established to describe the coupling between different physical fields within the power.

### 2.1. Mathematical Model of Electric–Thermal Field Coupling

SiC MOSFET continuously turns on and off during the conversion of electrical energy. The heat is generated due to switching loss and conducted through the physical layers of the device from the chip to the copper substrate, resulting in temperature changes in each layer. The temperature change will cause changes in the conductivity of the chip and bonding line, which will affect the heat generation of the device. Thus, relationships between electrical and thermal properties can be expressed as follows:

(1) $$\begin{array}{c} q=\left[\Pi \right]\cdot J-\left[\lambda \right]\cdot \nabla T \end{array}$$

(2) $$\begin{array}{c} J=\left[\sigma \right]\cdot \left(E-\left[\alpha \right]\cdot \nabla T\right) \end{array}$$

that

(3) $$\begin{array}{c} q=\left[\Pi \right]\cdot \left(\left[\sigma \right]\cdot \left(E-\left[\alpha \right]\cdot \nabla T\right)\right)-\left[\lambda \right]\cdot \nabla T \end{array}$$

where q is the heat generation rate per unit volume; $\left[\Pi \right]$ is the Peltier posting coefficient matrix; J is the current density; $\left[\lambda \right]$ is the thermal conductivity matrix; ∇T denotes the temperature gradient; [σ] is the electrical conductivity matrix; E is the electric field strength; and [α] is the Selkirk coefficient matrix.

### 2.2. Mathematical Model of Thermal–Moisture Field Coupling

Some packaging materials used in SiC MOSFET are hygroscopic. Changes in either the internal or external temperature of the device will affect the moisture distribution within it. The temperature dependence of this moisture transfer process is described by vapor saturation pressure $P_{sat}$:

(4) $$\begin{array}{c} G_{evap}=\frac{\partial \left(\varepsilon_{p}\rho_{g}W\left(1-s_{l}\right)\right)}{\partial t}+\rho_{g}u_{g}\cdot \nabla W+\nabla \cdot \left(-\rho_{g}D_{eff}\nabla W\right) \end{array}$$

which:

(5) $$\begin{array}{c} W=\frac{M_{v}\emptyset_{w}SP_{sat}\left(T\right)\varphi }{\rho_{g}} \end{array}$$

where $G_{evap}$ is the evaporation rate of the water vapor source; $\varepsilon_{p},\rho_{g}$ and $s_{l}$ represent porosity, wet air density, and liquid water saturation, respectively; $u_{g}$ is the wet air velocity; $M_{v}{,\emptyset }_{w}$ and $P_{sat}\left(T\right)$ are the molar mass of water, relative moisture, and vapor saturation pressure, respectively; S is the solubility of the material in water under given conditions; φ is relative humidity, W is the water content; $D_{eff}$ is the effective diffusivity in unsaturated media; and ∇ is the gradient operator.

The change in material properties caused by the diffusion of water vapor is represented by the change in effective thermal conductivity as well as the heat source:

(6) $$\begin{array}{c} \left(\varepsilon_{p}\left(s_{g}\rho_{g}C_{p,g}+s_{l}\rho_{l}C_{p,l}\right)+\theta_{s}\rho_{s}C_{p,s}\right)\frac{\partial T}{\partial t}+\nabla \cdot \left(-k_{eff}\nabla T\right)=Q_{1}+Q_{evap} \end{array}$$

which:

(7) $$\begin{array}{c} k_{eff}=\varepsilon_{p}\left(s_{g}k_{g}(W)+s_{l}k_{l}\right)+\theta_{s}k_{s}(W) \end{array}$$

(8) $$\begin{array}{c} Q_{evap}=L_{v}G_{evap} \end{array}$$

where $\varepsilon_{p}$ is the porosity; the subscripts g,s, and l are the gas, solid, and liquid phases, respectively; ρ is the density; $C_{p}$ is the constant pressure heat capacity; θ denotes the content; $k_{eff}$ is the effective thermal conductivity; k is the thermal conductivity; W is the water content; Q is the enthalpy flux due to water vapor diffusion and the capillary flux due to liquid water; $Q_{evap}$ is the total latent heat source in the domain; $L_{v}$ is the latent heat of evaporation; and ∇ is the gradient operator.

### 2.3. Mathematical Model of Thermal–Stress Field Coupling

The existence of a temperature gradient inside the device causes thermal stress and thermal expansion, both of which will affect the strain situation inside the device, and the thermoelasticity theory can be described as:

(9) $$\begin{array}{c} \varepsilon_{T}=\left[\begin{array}{c} \varepsilon_{Tx}\\ \varepsilon_{Ty}\\ \varepsilon_{Tz} \end{array}\right]=\frac{1}{e}\left[\begin{array}{ccc} 1 & -\mu & -\mu \\ -\mu & 1 & -\mu \\ -\mu & -\mu & 1 \end{array}\right]\left[\begin{array}{c} \sigma_{Tx}\\ \sigma_{Ty}\\ \sigma_{Tz} \end{array}\right]+\alpha \left[\begin{array}{c} {∆T}_{x}\\ {∆T}_{y}\\ {∆T}_{z} \end{array}\right] \end{array}$$

where $\varepsilon_{T}$ is the thermal strain; x,y,z are three normal directions; e is the elastic modulus; $\sigma_{T}$ is the thermal stress; ∆T is the temperature change at different moments; and α is the coefficient of the thermal expansion of the material.

### 2.4. Mathematical Model of Moisture–Stress Field Coupling

As moisture expansion has a similar effect on material as thermal expansion, the relationship between hygroscopic stress is established by applying the temperature effect theory to the material. Assuming no stresses or strains are generated during the expansion process, the strain induced by the moisture change can be expressed as:

(10) $$\begin{array}{c} \varepsilon_{W}=\left[\begin{array}{c} \varepsilon_{Wx}\\ \varepsilon_{Wy}\\ \varepsilon_{Wz} \end{array}\right]=\frac{1}{e}\left[\begin{array}{ccc} 1 & -\mu & -\mu \\ -\mu & 1 & -\mu \\ -\mu & -\mu & 1 \end{array}\right]\left[\begin{array}{c} \sigma_{Wx}\\ \sigma_{Wy}\\ \sigma_{Wz} \end{array}\right]+\beta \left[\begin{array}{c} {∆W}_{x}\\ {∆W}_{y}\\ {∆W}_{z} \end{array}\right] \end{array}$$

where $\varepsilon_{W}$ is the moisture strain; x,y,z are three normal directions; e is the modulus of elasticity; $\sigma_{W}$ is the moisture stress; ∆W is the moisture gain; and β is the moisture expansion coefficient of the material.

### 2.5. Mathematical Model of Thermal–Moisture Stress

The stress and strain of the device under the influence of temperature and humidity result from changes in temperature and humidity within the material. Thermoelastic mechanics principles are employed to calculate the stress and strain of the device during exposure to temperature and humidity. The thermo-hydroelastic equation can be expressed as follows:

(11) $$\begin{array}{c} \left[\begin{array}{c} {∆\varepsilon }_{x}\\ {∆\varepsilon }_{y}\\ {∆\varepsilon }_{z} \end{array}\right]+\frac{1}{1-2\mu }\left[\begin{array}{c} \sigma_{x}\\ \sigma_{y}\\ \sigma_{z} \end{array}\right]=\frac{2\left(1+\mu \right)}{e}\left(C_{sat}\cdot \beta \left[\begin{array}{c} W_{x}\\ W_{y}\\ W_{z} \end{array}\right]+\alpha \left[\begin{array}{c} T_{x}\\ T_{y}\\ T_{z} \end{array}\right]\right) \end{array}$$

where Δ is the Laplace operator; x,y,z are three normal directions; ε is the strain component; σ is the stress; μ is the Poisson ‘s ratio of the material; e is the elastic modulus of the isotropic material; $C_{sat}$ is the saturated moisture content of the material; α and β are the thermal and moisture expansion coefficients of the material; and W and T are the water content and temperature, respectively.

### 2.6. Mathematical Model of Thermal–Moisture Stress–Vibration Field Coupling

During an aircraft’s flight, the power device can be affected by mechanical vibration due to changes in acceleration and deceleration. Its motion equation is:

(12) $$\begin{array}{c} \left[M\right]\{\ddot{X}\}+\left[C\right]\{\dot{X\}}+\left[K\right]\{X\}=\{F_{\left(t\right)}\} \end{array}$$

where [M] is the mass matrix; [C] is the damping matrix; [K] is the structural stiffness matrix; $\left\{F_{\left(t\right)}\right\}$ is the load vector; and $\left\{X\right\},\left\{\dot{X}\right\},$ and $\left\{\ddot{X}\right\}$ are the displacement, velocity, and acceleration matrices in the x,y,z directions, respectively.

For a damped system, the eigenfrequency is $\omega_{d}=\sqrt{\frac{K}{M}}\sqrt{1-\xi^{2}}$, where ξ is the damping coefficient.

Under the influence of temperature and moisture, the device experiences stress and strain not only from mechanical vibrations but also due to variations in temperature and humidity within the material. The stress and strain caused by mechanical vibrations are determined using principles of elastic mechanics, while the stress and strain resulting from changes in temperature and humidity are calculated using thermoelastic theory. The mathematical model for thermal–moisture stress and vibration is further analyzed using Equations (11) and (12).

(13) $$\begin{array}{c} \left[\begin{array}{c} {∆\varepsilon }_{x}\\ {∆\varepsilon }_{y}\\ {∆\varepsilon }_{z} \end{array}\right]+\frac{1}{1-2\mu }\left[\begin{array}{c} \sigma_{x}\\ \sigma_{y}\\ \sigma_{z} \end{array}\right]+\frac{2\left(1+\mu \right)}{e}\left[\begin{array}{c} F_{\left(t\right)x}\\ F_{\left(t\right)y}\\ F_{\left(t\right)z} \end{array}\right]=\frac{2\left(1+\mu \right)}{e}\left(C_{sat}\cdot \beta \left[\begin{array}{c} W_{x}\\ W_{y}\\ W_{z} \end{array}\right]+\alpha \left[\begin{array}{c} T_{x}\\ T_{y}\\ T_{z} \end{array}\right]\right) \end{array}$$

### 2.7. Multi-Stress Coupling Mathematical Model of SiC MOSFET

According to the mathematical model presented in Equations (1)–(13), we further obtain the multi-stress field coupling mathematical model of SiC MOSFET.

(14) ∆εx∆εy∆εz+11−2μσxσyσz+21+μeFtxFtyFtz=21+μeCsat·βWxWyWz+αTxTyTzM{X¨}+C{X}˙+K{X}={Ft}Gevap=∂εpρgW1−sl∂t+ρgug·∇W+∇·−ρgDeff∇Wq=Π·σ·E−α·∇T−λ·∇T

**Figure 1:**
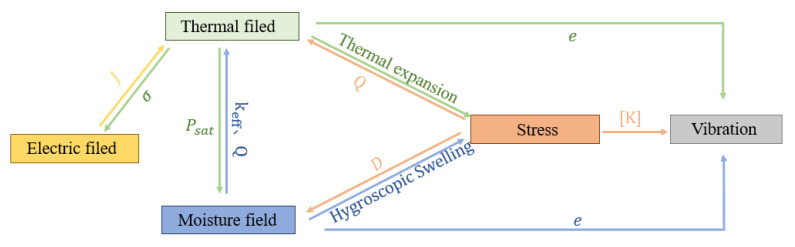
Coupling relationship between fields. Where J is the current density; σ is the conductivity; $P_{sat}$ is the vapor saturation pressure; $k_{eff}$ is the effective thermal conductivity; Q is the heat source; D is the diffusion coefficient; e is the modulus of elasticity; and [K] is the structural stiffness matrix.

The influence relationship between the fields in the mathematical modeling process is shown in Figure 1.

Under the action of electrical signals, heat is generated due to loss. Changes in junction temperature will, in turn, affect the chip conductivity. At the same time, changes in the internal temperature of the module will affect the water vapor saturation pressure, which will lead to changes in the saturated moisture content of the encapsulated material, affecting the humidity distribution within the module. As the moist air invades the device, the mode of heat conduction in the material changes from a single solid phase to a coexistence of solid, liquid, and gas. With the accumulation of time, the water content inside the module continues to increase, resulting in a certain change in the effective thermal conductivity and heat. The uneven distribution of temperature and humidity in various encapsulation materials, as well as the mismatched thermal and humidity expansion coefficients, can cause module deformation due to expansion. This deformation subsequently impacts the heat conduction and humidity transfer processes within the module. Temperature and humidity can impact the Young’s modulus of the material, consequently affecting the vibration mode and frequency of the devices, leading to variations in device stress.

## 3. COMSOL Modeling of SiC MOSFET

### 3.1. SiC MOSFET Model Building

**Figure 2:**
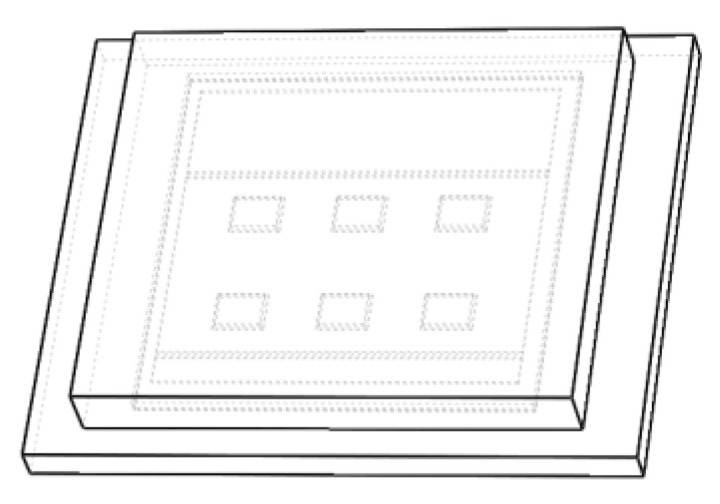
Three-dimensional model of SiC MOSFET.

In Figure 2, the physical model of C3M0016120K SiC MOSFET from CREE company is established. To simplify the analysis, the bonding lines are not initially considered. The device consists of eight layers: potting adhesive, chip (including passivation layer), chip solder layer, DBC upper copper layer, ceramic layer, DBC lower copper layer, substrate solder layer, and substrate. The model is designed using SOLIDWORKS and then imported into COMSOL to build the environment, with relevant parameters referenced in [12,14,15,16,17,18,19,20].

### 3.2. SiC MOSFET Model Loads and Boundary Conditions

Referring to the electrical parameters in the C3M0016120K datasheet, the gate voltage value is set to 15 V, the drain current amplitude is 115 A, and the source is grounded in the electric field simulation. The electrical conduction of bonding wires is ignored, so the upper surface of the chips is grounded.

The heat generated internally by the device due to electricity is transferred through heat conduction from the chip, passing through various physical layers and reaching the copper substrate. The copper substrate then transfers heat to the radiator. Forced heat dissipation is typically used in the device. To enhance simulation convergence and the heat dissipation effect of the equivalent radiator, the convective heat transfer coefficient on the lower surface of the copper substrate is set to 5000 $W/(m^{2}\cdot K)$ and the emissivity of the remaining surface to the environment is set to 0.5 because of the large volume difference between SiC MOSFET and the matching radiator. The temperature of the surrounding environment is set at 298.15 K. In practical use, screw positions are set as rigid connectors to restrict their rotation and movement.

To simulate water vapor diffusion behavior, one surface of the air domain is set as an inflow interface with a flow rate of 0.2 m/s, while the other surfaces are open boundaries to simulate the flow of wet air during operation.

## 4. Degradation Mechanism Analysis of SiC MOSFET Based on Multi-Stress Coupling Model

### 4.1. Degradation Mechanism of Electric–Thermal–Stress Coupled Model

To realistically simulate the switching process of SiC MOSFET, the drain current is set as a rectangular waveform with a switching time of 1 × 10^−18^ s and a duty cycle of 50%.

**Figure 3:**
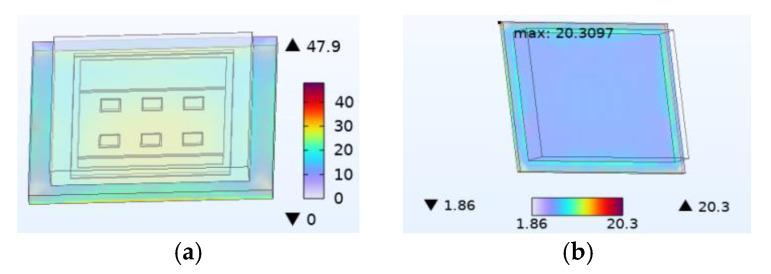
Thermal stress distribution diagram (MPa). (**a**) The SiC device thermal stress distribution diagram. (**b**) Chip solder layer thermal stress distribution diagram.

Figure 3a shows the thermal stress distribution during device operation. It can be observed that the stress is mainly distributed in the chip solder layer, the copper layer on the DBC, the connection between the DBC solder layer and the copper substrate, and the bottom constraint of the substrate. The upper copper layer experiences maximum stress because of the heat generated by all chips being transmitted to the copper substrate through it and its irregular shape. The thermal stress does not affect device failure due to the high yield strength of copper. However, solder has a much smaller yield strength compared with copper, making the chip solder layer more susceptible to failure owing to its mechanical viscoplastic effect. Figure 3b shows the distribution of stress in the chip solder layer. The maximum stress value occurs at the corner of the back side and is 20.3 MPa.

**Figure 4:**
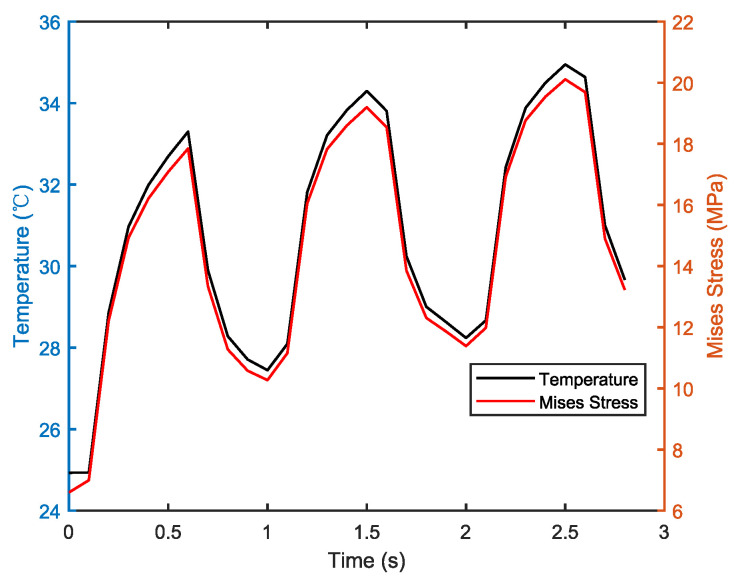
Chip solder layer temperature and stress average curve.

The thermal stress in the solder layer follows a similar pattern as that of temperature changes. As shown in Figure 4, the thermal stress increases with rising temperature when the device is turned on and decreases with temperature after turning off. Due to long-term device operation under the influence of electrical signals, heat accumulates inside the device. When the thermal stress reaches a certain level, cracks start forming at the corners of the solder layer where it comes into contact with the copper layer and chip. The cracks then gradually spread to the interior of the solder layer, resulting in the peeling of the solder layer and the failure of normal heat conduction, which ultimately leads to chip failure. The root cause of solder layer failure is the mismatch in CTE between the solder layer, silicon carbide chip, and the upper copper layer.

**Figure 5:**
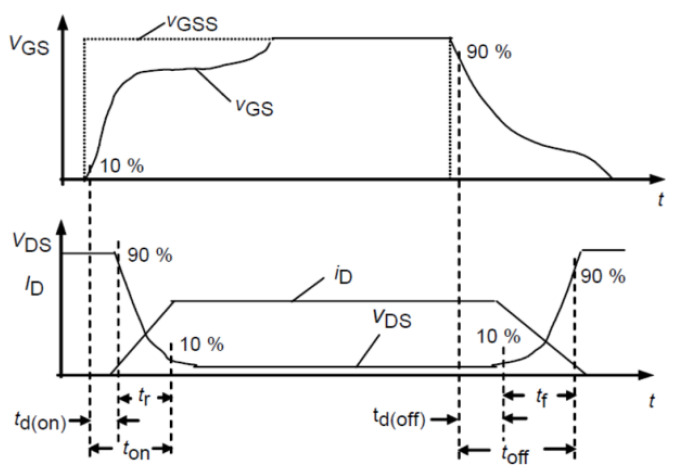
Switching times definition.

**Figure 6:**
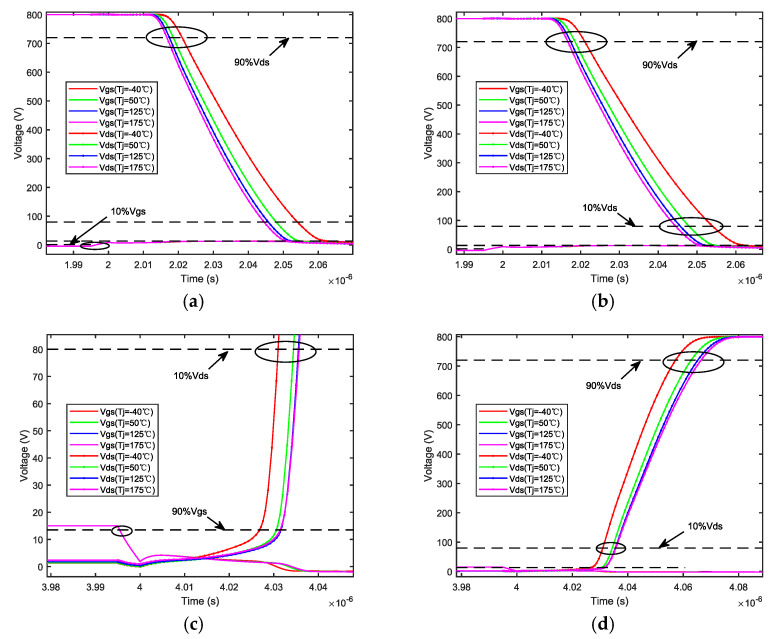
Switching time at different junction temperatures. (**a**) Turn−on delay time. (**b**) Rise time. (**c**) Turn−off delay time. (**d**) Fall time.

The thermal effect affects not only the device packaging but also the electrical parameters of the chip. The definition of the switching time in Figure 5 and Figure 6 gives the variation in switching times under different junction temperatures. The analysis results indicate that as the junction temperature increases, the turn-on delay time becomes shorter and the rise time decreases, and then the turn-on time is shortened. Conversely, the turn-off delay time becomes longer, the fall time increases, and then the turn-off time is increased. These changes are attributed to temperature affecting the parasitic capacitance of the chip.

### 4.2. Degradation Mechanism of Electric–Thermal–Moisture Coupled Model

Wet air invades the device from outside to inside. Due to the poor hygroscopicity of the copper substrate, wet air is primarily concentrated within the silicone encapsulation layer. Since the moisture absorption rate of the encapsulation material is slower than that of heat conduction, only qualitative simulations are performed.

**Figure 7:**
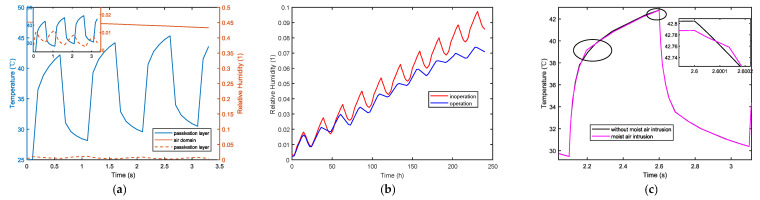
Interaction laws in thermal–moisture coupling fields. (**a**) Relative moisture and temperature change patterns with time. (**b**) The effect law of thermal–moisture coupling on relative moisture. (**c**) The effect law of thermal–moisture coupling on temperature.

Figure 7a shows changes over time in the temperature and relative moisture of the passivation layer, as well as the relative moisture in the air domain during device operation. Comparing the blue and red lines, it can be seen that the heat generated reduces the internal relative moisture content of the device when the chip is functioning. A small decrease in the relative moisture of the air domain occurs due to continuous heat dissipation from the device to the air, which leads to a gradual increase in the surrounding air temperature, subsequently causing the relative moisture to fluctuate.

The temperature and moisture of the external environment are atmospheric data from a particular location. Figure 7b shows changes in the relative moisture of the passivation layer. Similar to the results in Figure 7a, moisture fluctuation within the device is suppressed during device operation. This is because of the temperature gradient from inside to outside, while the temperature gradient from outside to inside caused by moisture fluctuation counteracts each other, thereby weakening the amplitude of relative moisture fluctuation resulting from external temperature fluctuation. Consequently, it leads to a reduction in relative moisture fluctuation within the device. Figure 7c shows the impact of thermal–moisture coupling on temperature. Although this coupling slightly causes a decrease in temperature overall, it also produces a certain fluctuation peak during the temperature change.

### 4.3. Degradation Mechanism of Electric–Thermal–Moisture–Stress Coupled Model

**Figure 8:**
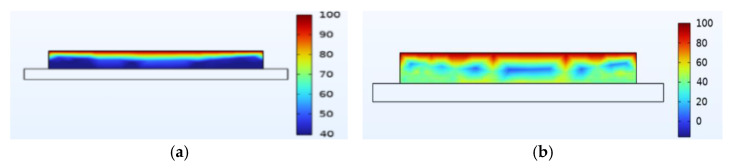
Silica gel layer concentration distribution (mol/m^3^). (**a**) Moisture field. (**b**) Thermal−moisture coupling field.

**Figure 9:**
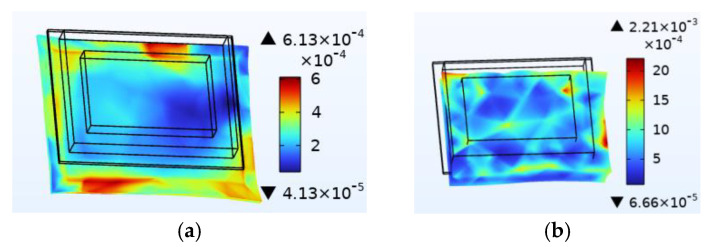
Moisture stress distribution (MPa). (**a**) Passivation layer. (**b**) Solder layer.

Just like thermal stress, moisture stress is also generated within hygroscopic material after environmental moisture air invades the device. To realistically simulate the process of moisture stress generation, the electric–thermal–moisture coupling model data are used to realize the electric–thermal–moisture–stress coupling. Figure 8 shows the concentration distribution of the silica gel layer under different fields. Comparing Figure 8a,b, it can be found that during wet air diffusion, the distribution of moisture content inside the device is non-uniform. Due to different deformation of the material under different humidity, a constraining force is generated. Figure 9 shows the moisture stress distribution of the solder layer and the passivation layer under the same humidity change. The maximum moisture stress of the passivation layer is 6.13 × 10^−4^ MPa, and that of the solder layer is 2.21 × 10^−3^ MPa. The diffusion behavior of wet air in two packaging layers is similar. However, the stress is different due to the coefficient of moisture expansion (CME) between the two packaging layers. Furthermore, significant warpage occurs at the corners of the passivation layer and chip solder layer under the effect of moisture stress, which may be related to the diffusion behavior of humid air from outside to inside. Similar to thermal stress, this phenomenon may cause the solder layer to peel off.

**Figure 10:**
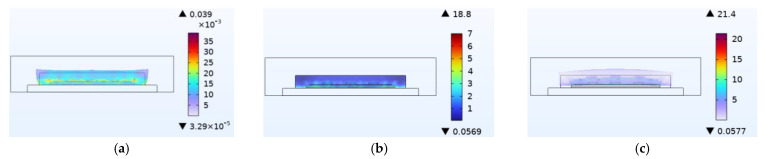
Analysis of stress results for the silica gel layer (MPa). (**a**) Thermal stress. (**b**) Moisture stress. (**c**) Stress after thermal−moisture coupling.

When wet air invades the device from outside to inside, the silicone layer is the first to be affected. Figure 10 shows stress results for the silicone layer under the same simulation conditions. Compared to thermal stress alone, coupling with wet air results in a stress increase of 2.6 MPa, which is 21.361 MPa higher than the stress caused by moisture alone. Similarly, compared to moisture stress alone, coupling with temperature results in a stress increase of 21.361 MPa, which is 2.6 MPa higher than that caused by thermal stress alone. Figure 7 has shown that moisture diffusion will slightly decrease the temperature inside the device, while chip operation also reduces the relative moisture inside the device. However, the combined effect increases stress on the device, thereby exacerbating device damage. This phenomenon occurs not only in the silica gel layer but also in other passivation layers and solder layers. This is because moisture changes the thermal time constant, which causes the fluctuation peak generated by temperature change to produce greater stress. Additionally, moisture diffusion causes hygroscopic material to expand and aggravate the stress.

### 4.4. Degradation Mechanism of Stress–Vibration Coupled Model

**Figure 11:**
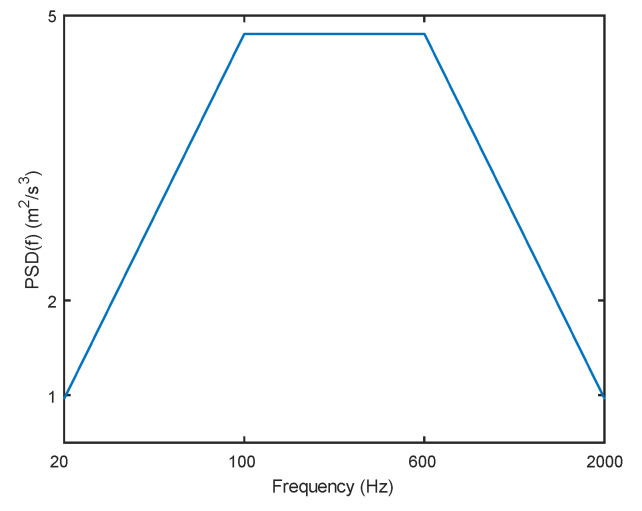
Acceleration power spectral density.

Apart from thermal and moisture stresses, mechanical vibrations can also influence the device’s reliability. During testing, the acceleration power spectral density is provided with reference to the random vibration requirement specified in RTCA/DO-160G, as shown in Figure 11, and the device is loaded using the power spectral density. In building the coupling model of thermal–moisture stress and vibration, the model is first subjected to steady-state analysis to obtain temperature and moisture distribution for normal device operation. Subsequently, these temperature and moisture field conditions are applied as loads to the stress field for coupling analysis. As the solder layer is thin, the stress of the solder layer is mainly analyzed.

**Figure 12:**
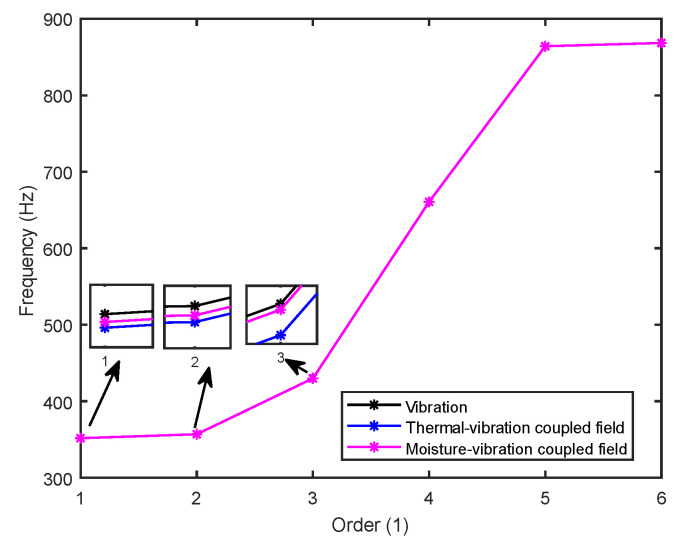
The natural frequencies of the device under different fields.

The stability of the structure is closely related to the previous order modes. The natural frequency and the first three order modes of the device under the combined influence of thermal–moisture stress and vibration are shown in Figure 12 and Table 1. The analysis results indicate that the intrinsic frequency of each order for the device decreases non-linearly and monotonically under the influence of temperature and moisture, and this effect is consistent with the vibration pattern. Moreover, the results show that the impact mechanism of heat and humidity on the device’s vibration characteristics is consistent, which is all due to the change in material performance leading to the change in the material stiffness matrix. However, the influence of temperature on the vibration characteristics of the device is greater than that of humidity. Under steady-state temperature and moisture load, the device’s deformation is comparable to that generated by random vibration loads. The first-order mode has the greatest influence on the chip solder layer, while deformation at the constraint is minimal. Therefore, vital components can be considered to be placed near the fixed point in the component distribution design.

Table 2 compares the stress distribution in the solder layer. Stress primarily concentrates at the corners of the junction between the chip and the solder layer, so the contact surface with the chip is more susceptible to failure. The maximum stress results indicate that the introduction of temperature and moisture during vibration significantly increases stress levels, with thermal stress being the main influencing factor. It has been shown in Figure 10 that the combined action of temperature and moisture will aggravate stress generation. Similarly, under the combined action of thermal–moisture stress and vibration, the device is more susceptible to failure.

## 5. Conclusions

The finite element method was used to simulate the SiC MOSFET’s operational environment. A multi-physical field coupling model was established, and the following conclusions were drawn on the multi-physical field coupling degradation mechanism:

1. Thermal stress is caused by uneven temperature distribution inside the device and mismatched CTEs of each package layer. Temperature changes will affect the parasitic capacitance, resulting in a shorter turn-on time but a longer turn-off time.
2. Moisture diffusion slightly decreases internal temperature, but it generates fluctuation peaks during temperature changes, and at the same time, the wet fluctuations inside the device will be suppressed when the device works.
3. Moisture stress is caused by uneven moisture distribution inside the device and mismatched CMEs of each package layer. In the thermal–moisture coupling field, moisture changes the thermal time constant. More stress is generated by temperature change-related fluctuation spikes. Additionally, the moisture expansion of moisture-absorbing materials can aggravate stress generation and then accelerate device failure.
4. The mechanism of thermal and moisture effects on the vibration characteristics of the device is consistent, which is all due to the change in material performance leading to the change in the material stiffness matrix. However, the influence of temperature on the vibration characteristics of the device is greater than that of humidity. Under the combined action of thermal–moisture stress and vibration, the device is more susceptible to failure.

In summary, the analysis results demonstrate that the proposed multi-physics coupling modeling method using COMSOL is reasonable and effective. The multi-stress coupling model can simulate the degradation process of SiC MOSFET more comprehensively and accurately, providing references for improving the reliability design of power device packaging.

## Figures and Tables

**Table 1:** Comparison of the first three order modes under different fields.

	Vibration	Thermal Stress and Vibration	Moisture Stress and Vibration	Thermal–Moisture Stress and Vibration
First-order vibration mode	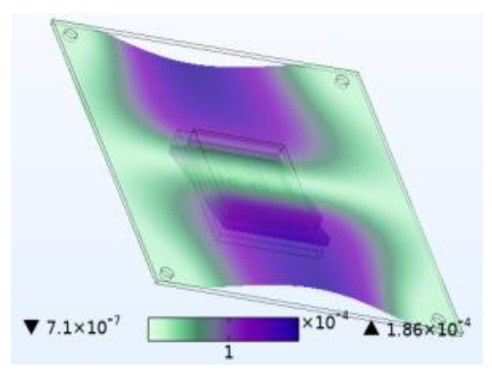	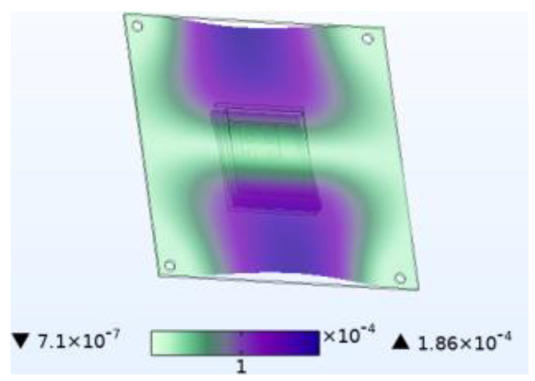	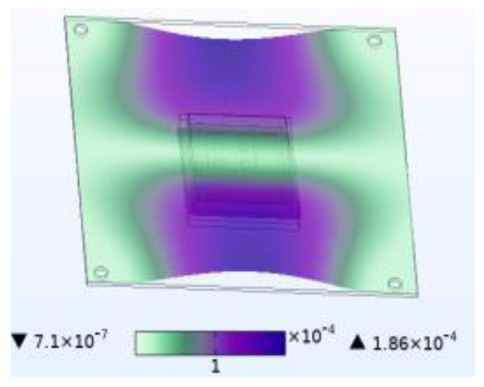	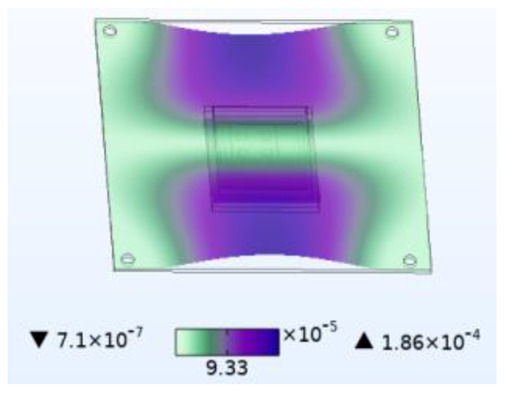
Second-order vibration mode	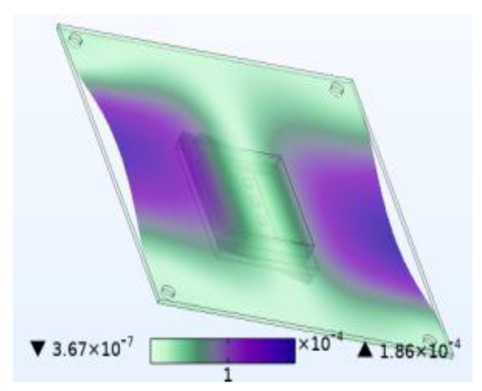	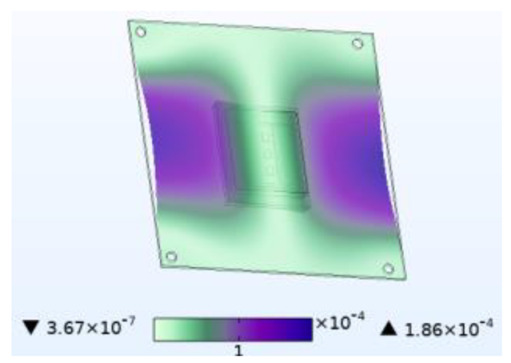	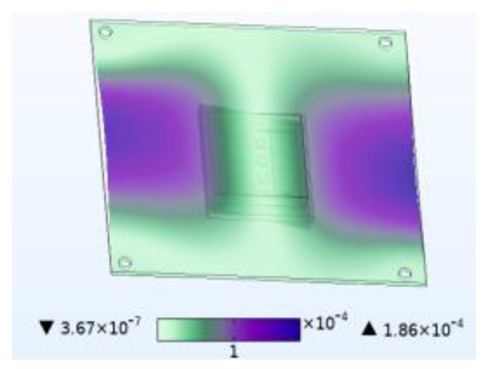	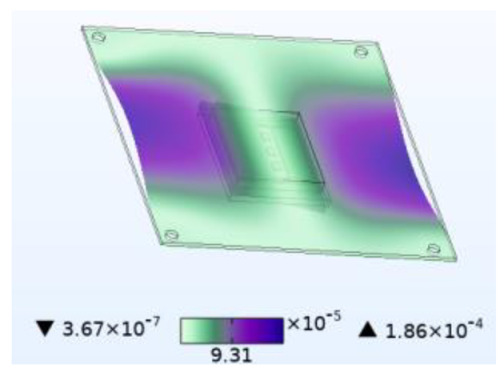
Third-order vibration mode	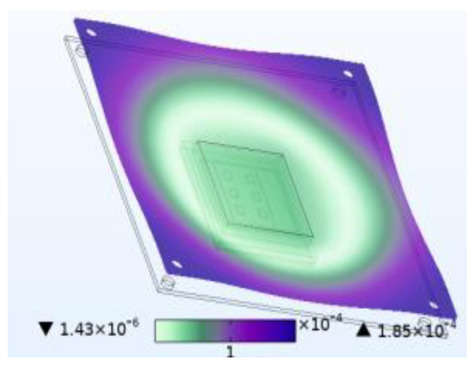	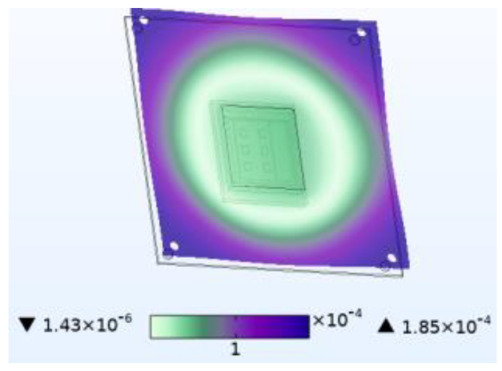	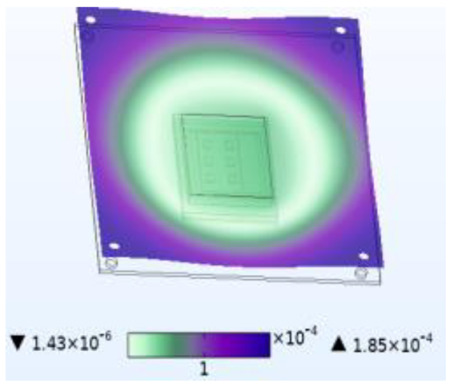	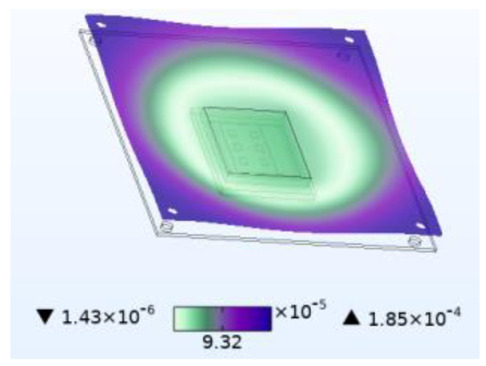

**Table 2:** Comparison of the Y-direction stress of the solder layer under different fields.

	Vibration	Thermal Stress and Vibration	Moisture Stress and Vibration	Thermal–Moisture Stress and Vibration	Thermal–Moisture Stress
Stress distribution	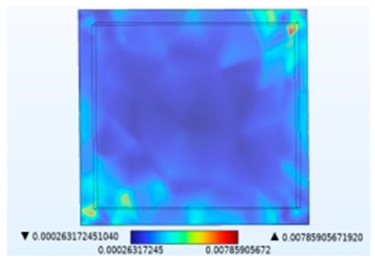	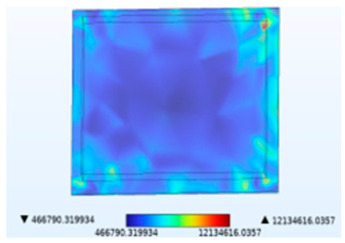	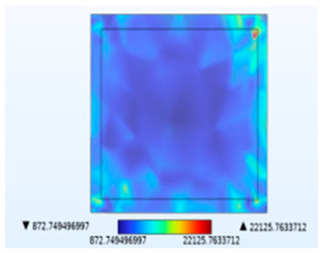	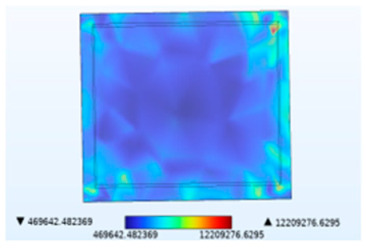	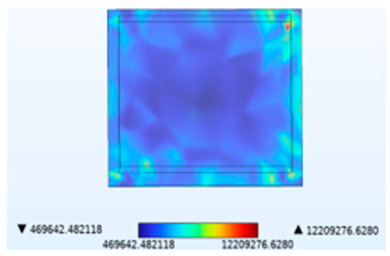
Maximum stress value (Pa)	0.00785905672	12,134,616.0357	22,125.7633712	12,209,276.6295	12,209,276.6280
